# Stanniocalcin 1 in Patients with Refractory Colorectal Cancer Treated with Regorafenib: A *Post Hoc* Biomarker Analysis of the TEXCAN and CORRECT Trials

**DOI:** 10.1158/2767-9764.CRC-24-0246

**Published:** 2025-02-11

**Authors:** Angélique Vienot, Dewi Vernerey, Adeline Bouard, Elodie Klajer, Stefano Kim, Christophe Tournigand, Christophe Louvet, Thierry André, Benoît Rousseau, Mylène Wespiser, Laurie Spehner, Ying A. Wang, Anke Weispfenning, Emmanuelle Dochy, Christophe Borg

**Affiliations:** 1 Department of Medical Oncology, University Hospital of Besançon, Besançon, France.; 2 University of Franche Comté, EFS, INSERM, UMR RIGHT, Besançon, France.; 3 Université Paris Est Créteil, Hôpital Henri-Mondor, AP-HP, Créteil, France.; 4 Institut Mutualiste Montsouris, Paris, France.; 5 Sorbonne Université and Hôpital Saint-Antoine, Paris, France.; 6 Memorial Sloan Kettering Cancer Center, New York, New York.; 7 Bayer HealthCare Pharmaceuticals, Cambridge, Massachusetts.; 8 Bayer AG, Berlin, Germany.; 9 Bayer SA-NV, Brussels, Belgium.

## Abstract

Biomarkers for antiangiogenic drugs in chemorefractory metastatic colorectal cancer (mCRC) are lacking. We investigated the relationship between stanniocalcin 1 (STC1) and outcomes in patients treated with regorafenib in the TEXCAN and CORRECT trials. Baseline plasma STC1 protein levels were measured by ELISA from patients with chemorefractory mCRC enrolled in TEXCAN (regorafenib *n* = 48) and CORRECT (placebo *n* = 211; regorafenib *n* = 435). The relationship between STC1 levels and overall survival (OS) was assessed using a Cox proportional hazards model. The median STC1 value was increased in patients with chemorefractory mCRC (1,211 pg/mL) compared with previously untreated patients (215 pg/mL). Using an optimized cut-off, STC1 was prognostic for OS [HR = 2.12, 95% confidence interval (CI), 1.79–2.50; *P* < 0.001], with a median OS of 7.63 months in the STC1-low group (<1,436.87 pg/mL; *n* = 400) and 3.81 months in the STC1-high group (≥1,436.87 pg/mL; *n* = 246). The interaction *P* value of lactate dehydrogenase and treatment revealed no predictive effect of lactate dehydrogenase levels on OS in terms of regorafenib (*P* = 0.598). A predictive analysis suggested a significant association between STC1 and regorafenib for OS (interaction *P* = 0.049). The median OS with regorafenib versus placebo was 8.32 versus 6.54 months in the STC1-low group (HR = 0.83, 95% CI, 0.66–1.03; *P* = 0.087) and 4.41 versus 3.09 months in the STC1-high group (HR = 0.64, 95% CI, 0.49–0.84; *P* = 0.001). Altogether, high STC1 protein levels have a predictive potential to characterize a population of patients with chemorefractory mCRC and poor prognosis in whom regorafenib has an increased level of efficacy.

**Significance:**

STC1 is a protein secreted by intratumor endothelial cells in which plasma concentrations increase in patients with chemorefractory mCRC. Based on analyses of patients with refractory mCRC in the TEXCAN and CORRECT trials, we found that STC1 plasma levels had a prognostic role for OS, with high levels associated with poor outcome. A predictive role for baseline STC1 levels was pointed out for regorafenib efficacy.

## Introduction

Most patients with metastatic colorectal cancer (mCRC) experience a relapse or progression of their disease following exposure to standard therapies, which typically involve treatment with fluoropyrimidines, oxaliplatin, irinotecan, bevacizumab, and EGFR-targeting antibodies (1). Despite improvements in clinical outcomes over the past decade, treatment of metastatic disease resistant to chemotherapy remains a challenge.

Regorafenib has immunomodulatory and antiangiogenic properties through the inhibition of multiple kinases, including the angiogenic kinases VEGFR, platelet-derived growth factor receptor, and tyrosine kinase with immunoglobulin and EGF homology domain 2 (TIE2; refs. 2–4). Regorafenib is approved for the treatment of mCRC in the refractory setting based on improvements in overall survival (OS) in the placebo-controlled, randomized, phase 3 CORRECT trial {median OS: 6.4 months with regorafenib vs. 5.0 months with placebo [HR = 0.77; 95% confidence interval (CI), 0.64– 0.94; one-sided *P* = 0.0052], which was subsequently confirmed in an Asian population of patients in the phase 3 CONCUR trial (5, 6). These results suggest that broad targeting of angiogenesis is an effective approach in the treatment of refractory mCRC.

Biomarker analysis of the CORRECT trial showed that regorafenib was consistently associated with clinical benefit in a range of patient subgroups based on both mutation status and protein biomarker concentrations, including that of angiogenic proteins (7). Defining the molecular mechanisms of disease progression and predictors of regorafenib efficacy remain important outstanding challenges in colorectal cancer.

Interaction between stromal and tumor cells is known to play a major role in the growth and progression of colorectal cancer (8). The angiogenic marker angiopoietin 2 (ANG2) has been identified as a potential prognostic biomarker for mCRC not previously treated with chemotherapy (9). ANG2 is expressed by endothelial cells at sites of vascular homeostasis and is involved in the regulation of angiogenesis through TIE2 and integrin signaling (10–12). Recent molecular characterization of the ANG2-related poor-prognosis colorectal cancer subset revealed that the ANG2-high colorectal cancer subgroup was not correlated with a specific consortium molecular subtype or genomic alterations. Instead, the subgroup was characterized by the expression of genes involved in angiogenesis, stromal organization, and invasion, one of which was stanniocalcin 1 (STC1; ref. 8).

STC1 is a secreted glycoprotein that is aberrantly expressed in a number of tumor types and acts in an autocrine/paracrine manner to promote tumor cell viability, proliferation, solid tumor invasion, and metastasis (13, 14). Expression of STC1 is modulated by hypoxia, resulting in tolerance to hypoxic conditions through reprogramming of tumor metabolism (the Warburg effect), reduction in reactive oxygen species, and antiapoptotic effects (15, 16). STC1 also promotes tumorigenicity and invasiveness through the upregulation of matrix metalloproteinase 9 and participation in epithelial–mesenchymal transition (16, 17). In summary, STC1 is a potential regulator of cancer cell and stroma interactions.

We hypothesized that STC1 expression reflected the presence of a complex colorectal cancer microenvironment in which cross-talk between angiogenesis-related signaling and stromal activation operates. In this *post hoc* analysis, we investigated STC1 in plasma as a potential prognostic biomarker in patients with refractory mCRC in the TEXCAN and CORRECT trials and as a potential predictive biomarker for regorafenib efficacy in the placebo-controlled randomized CORRECT trial.

## Materials and Methods

### Participants and treatment

Plasma samples from healthy volunteers and patients with colorectal cancer enrolled in the EPITOPES-CRC02, TEXCAN, and CORRECT clinical trials were analyzed for STC1 protein levels. Plasma from healthy volunteers was used to determine the normal distribution of STC1 protein values in tumor-free donors. Plasma from the EPITOPES-CRC02 study was used to characterize STC1 levels in untreated patients with mCRC. The TEXCAN and CORRECT trials included patients with chemotherapy-resistant mCRC. An exploratory investigation was performed using samples from the TEXCAN study to determine which plasma-related biomarker might influence the OS of patients with chemotherapy-resistant mCRC. Samples from the CORRECT study were used to determine the optimized cut-off and correlations between STC1 levels and relevant clinical/biological parameters. Prognostic validation and predictive analyses were performed using data from CORRECT.

For healthy volunteers, blood cells were collected in an anonymous manner (EFS, Bourgogne-Franche-Comté, France) as apheresis kit preparation after obtaining informed consent and according to EFS guidelines. The major exclusion criteria for blood collection were minors (<18 years), age more than 65 years, dehydration, fatigue, low levels of hemoglobin (>120 g/L for women and 130 g/L for men), flu symptoms, human immunodeficiency virus, human T-cell lymphotropic virus, or hepatitis B/C-positive status, autoimmune diseases, surgical procedures in the last 4 months, and vaccination in the last 4 weeks.

EPITOPES-CRC02 (NCT02817178) was a French multicenter prospective cohort study that aimed to determine the prognostic value of peripheral immune profiles in patients with previously untreated mCRC. Among the secondary objectives, this study sought to identify prognostic biomarkers in plasma and characterize their relationship with the tumor microenvironment. Patients from nine centers were enrolled between 2013 and 2016. Eligible patients were more than 18 years of age, with Eastern Cooperative Oncology Group (ECOG) performance status of 0 to 2, histologically proven colorectal cancer, were candidates for first-line chemotherapy with or without targeted therapy (antiangiogenic or anti-EGFR), and were enrolled more than 6 months after the end of adjuvant chemotherapy. Surgery of the primary tumor prior to initiation of chemotherapy was permitted.

Details of the single-arm, phase II TEXCAN study (NCT02699073) have been previously published (18, 19). Same inclusion criteria as for CORRECT were used (5). Briefly, patients with mCRC previously treated with, or who were not considered candidates for, standard therapies were treated with regorafenib 160 mg/day for 3 weeks on/1 week off. The primary endpoint was 2-month overall response rate by RECIST version 1.1, Choi, and modified Choi criteria. The main outcome was OS, measured from the first dose of regorafenib to death from any cause. Plasma samples were collected at baseline and stored at −20°C.

Details of the randomized, placebo-controlled, phase 3 CORRECT trial (NCT01103323) have been previously published (5). Briefly, patients with mCRC who had received all approved standard therapies (including a fluoropyrimidine, oxaliplatin, irinotecan, bevacizumab, and, if appropriate, an anti-EGFR antibody) were randomized to receive oral regorafenib 160 mg or placebo once daily for the first 3 weeks of each 4-week cycle. The primary endpoint was OS (time from randomization to death from any cause). Plasma was collected at baseline and stored at −80°C.

Written informed consent was obtained for all patients, and all protocols were approved by each center’s Institutional Review Board or independent Ethics Committee and followed the guiding principles of the Declaration of Helsinki. All participants with informed consent who had samples available were included in this analysis.

### Sample analysis

In the EPITOPES-CRC02, TEXCAN, and CORRECT trials, blood samples were drawn at baseline and immediately processed for plasma freezing. ELISA was used to measure ANG2 (Diaclone) and STC1 (R&D Systems) protein levels in plasma samples collected at baseline, according to the manufacturer’s instructions. Analyses of lactate dehydrogenase (LDH) levels were performed in each investigation center according to local techniques and reported as normal or above the upper limit of normal values according to local reference values. Each sample was analyzed in duplicate. Robustness was tested by repeating measures of a similar batch of control plasma in all experiments. Experiments were carried out in a blinded fashion.

### Gene expression analysis

Single-cell RNA sequencing data were collected from Gene Expression Omnibus accession number GSE178318 in order to determine the cell subsets expressing STC1 in colorectal cancer metastases. Data were processed with Seurat 4.0.6.

### Statistical analysis

The primary objective of this exploratory analysis was to assess the prognostic effect of STC1 in patients with chemorefractory colorectal cancer. The secondary objective was to define the relative efficacy of regorafenib according to STC1 values.

In CORRECT, all randomized patients who received at least one dose of study drug and who did not cross over from regorafenib to placebo were included. Across studies, only patients and participants with available biomarker values were included in the biomarker analysis sets.

Patient characteristics and STC1 protein levels were summarized using descriptive statistics. A Wilcoxon rank-sum test was performed for comparison of STC1 protein level distribution between study cohorts. The optimized cut-off of baseline plasma STC1 levels was determined based on the HR of a Cox proportional hazards model for OS combining both arms of CORRECT. A predictive and prognostic analysis was conducted, investigating a potential difference in treatment benefit between STC1-low and -high groups (predictive) and comparing OS between STC1-low and -high groups independent of the treatment received (prognostic). The interaction for the treatment effect on OS and the STC1-low and -high values were modeled by adding in a single Cox model the studied subgroups based on STCI levels, the treatment arm (regorafenib vs. placebo) parameters, and their interaction term.

OS was estimated using the Kaplan–Meier method, described using median with 95% CIs, and compared using the log-rank test. The Cox proportional hazards model was performed to estimate the HR and 95% CI.

Additionally, a multivariable Cox proportional hazards model was calculated to investigate the association between OS, STC1, and relevant clinical parameters in combination. The performance of the Cox model was evaluated using 10-fold cross-validation with 200 repetitions.

All analyses were performed using R software version 4.0.2 (R Development Core Team; http://www.r-project.org). Values of *P* < 0.05 were considered statistically interesting, and all tests were two-sided. Due to the *post hoc* nature of the study, *P* values were not corrected for multiple testing and should be considered exploratory.

### Data availability

Availability of data from CORRECT will be determined according to Bayer’s commitment to the European Federation of Pharmaceutical Industries’ Associations/Pharmaceutical Research and Manufacturers of America “Principles for responsible clinical trial data sharing.” This pertains to the scope, timepoint, and process of data access. As such, Bayer commits to sharing upon request from qualified scientific and medical researchers’ patient-level clinical trial data, study-level clinical trial data, and protocols from clinical trials in patients for medicines and indications approved in the United States and European Union as necessary for conducting legitimate research. All other patient-level data generated from this study are not publicly available due to patient privacy requirements but are available upon reasonable request from the corresponding author.

## Results

### STC1 plasma protein levels are increased in patients with chemotherapy-refractory mCRC

STC1 protein levels were measured in the plasma of healthy volunteers (*n* = 66), in patients with mCRC at diagnosis (EPITOPES-CRC02 study; *n* = 156), and in the chemotherapy-refractory setting (CORRECT study; *n* = 646). Median levels of STC1 were 123 pg/mL (range: 0–692) in healthy volunteers and 215 pg/mL (range: 0–4,750) in patients with previously untreated mCRC. By contrast, increased STC1 levels with a median of 1,211 pg/mL (range: 320–6,568) were observed in patients with chemotherapy-refractory mCRC from the CORRECT study (Table 1). These results suggest that STC1 protein levels in plasma are elevated in patients with advanced disease and those who are refractory to chemotherapy (Wilcoxon rank-sum test <0.001).

**Table 1:** STC1 plasma protein expression in healthy donors and patients with previously untreated mCRC (EPITOPES-CRC02) and in the refractory setting (CORRECT)

Study and patient population	*N*	STC1 (pg/mL)	
		Mean (SD)	Median (range)
Healthy volunteers	66	237 (197)	123 (0–692)
EPITOPES-CRC02 study: first-line mCRC	156	420 (640)	215 (0–4,750)
CORRECT study: third-line or later mCRC	646	1,473 (941)	1,211 (320–6,568)

We took advantage of a previously reported single-cell atlas of colorectal cancer liver metastases to investigate the pattern of STC1 expression (20). t-distributed stochastic neighbor embedding analyses revealed a selective expression of STC1 in a single cluster (Supplementary Fig. S1A). STC1-producing cells are included in the cluster of endothelial cells characterized by claudin 5 expression (Supplementary Fig. S1B). This analysis showed that STC1 expression is mainly located within endothelial cells, suggesting that STC1 production is more related to endothelial cell activation in advanced colorectal cancer disease rather than to the number of tumor cells.

### STC1 but not ANG2 is a potential prognostic biomarker in patients with chemorefractory mCRC in the TEXCAN trial

**Figure 1:**
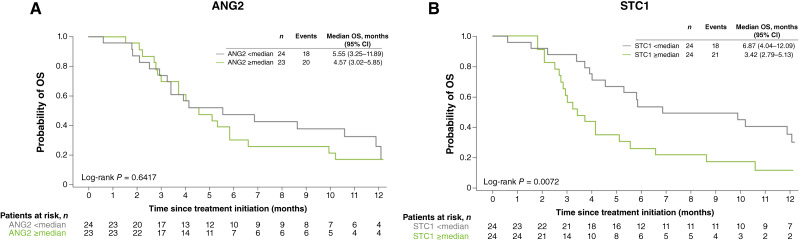
OS in patients with refractory mCRC from the TEXCAN study according to baseline plasma protein levels of (**A**) ANG2 and (**B**) STC1.

As a correlation between STC1 and ANG2 levels has been previously demonstrated (8) and both are expressed in endothelial cells, we next decided to investigate their prognostic potential in patients with colorectal cancer. For this exploratory analysis, STC1 protein level measurements were obtained from 48 of the 55 patients with mCRC resistant to chemotherapy and treated with regorafenib in the TEXCAN study (18). Median STC1 levels were 690 pg/mL (range: 135–1,390). Although there was no correlation between OS and plasma levels of ANG2 (log-rank *P* = 0.6417), patients with high STC1 levels (≥median) were at greater risk of death (median OS of 3.42 months) than patients with lower levels (median OS of 6.87 months; log-rank *P* = 0.0072; Fig. 1A and B). These results suggest that STC1 may be a prognostic biomarker in patients with mCRC resistant to chemotherapy and eligible for regorafenib.

### Validation analysis of the prognostic value of STC1 in patients with refractory mCRC in the CORRECT trial

In order to validate these results in a randomized dataset, we analyzed the prognostic value of STC1 in patients with treatment-refractory mCRC who received either regorafenib or placebo in the CORRECT trial. Of 760 patients enrolled in CORRECT (5), plasma STC1 protein level measurements were obtained from 646 patients (regorafenib *n* = 435; placebo *n* = 211). STC1 protein levels were comparable between the regorafenib (median: 1,181 pg/mL; range: 375–6,568) and placebo (median: 1,233 pg/mL; range: 320–6,223) cohorts (Supplementary Table S1; Supplementary Fig. S2A; Wilcoxon rank-sum test *P* = 0.7).

Using the patient population from CORRECT, an optimized cut-off of baseline plasma STC1 protein levels at 1,436.87 pg/mL was determined to allow best discrimination of the risk of death for patients with mCRC in this patient population (Supplementary Fig. S2B). Of note, only 7% of untreated mCRC from the EPITOPES-CRC02 study had STC1 levels above 1,400 pg/mL, suggesting that STC1 might be a biomarker related to the chemorefractory setting.

The baseline characteristics of patients in the STC1-low (<1,436.87 pg/mL; *n* = 400) and STC1-high (≥1,436.87 mg/mL; *n* = 246) groups were generally consistent with those of the overall cohort, except for liver metastases (Table 2). There was no difference in age, sex, ECOG performance status, and *RAS* mutations between patients with low and high STC1 protein levels. By contrast, a Fisher exact test indicated a difference in STC1 protein levels (low vs. high) and the distribution of liver metastases at baseline (*P* < 0.001).

**Table 2:** Baseline characteristics of the overall CORRECT cohort and STC1 subgroups (optimized cut-off^a^)

	STC1-low group	STC1-high group	Total
	(*n* = 400)	(*n* = 246)	(*N* = 646)
Age, median (range), years	61 (22–85)	61 (25–82)	61 (22–85)
Male, *n* (%)	245 (61)	153 (62)	398 (62)
ECOG PS, *n* (%)			
0	242 (61)	113 (46)	355 (55)
1	158 (40)	133 (54)	291 (45)
Primary site of disease, *n* (%)			
Colon	249 (62)	169 (69)	418 (65)
Rectum	127 (32)	62 (25)	189 (29)
Colon and rectum	23 (6)	15 (6)	38 (6)
Missing	1 (<1)	0	1 (<1)
Number of previous treatment lines for metastatic disease, *n* (%)			
≤3	221 (55)	113 (46)	334 (52)
>3	179 (45)	133 (54)	312 (48)
Liver metastases, *n* (%)			
No	152 (38)	25 (10)	177 (27)
Yes	248 (62)	221 (90)	469 (73)
*KRAS* mutation^b^, *n* (%)			
No	145 (36)	112 (46)	257 (40)
Yes	239 (60)	121 (49)	360 (56)
Missing	16 (4)	13 (5)	29 (4)

Abbreviations: ECOG PS, ECOG performance status.

a Optimized STC1 plasma levels: STC1 low: <1,436.87 pg/mL; STC1 high: ≥1,436.87 pg/mL.

b *KRAS* mutation status was based on historic mutation analysis.

**Figure 2:**
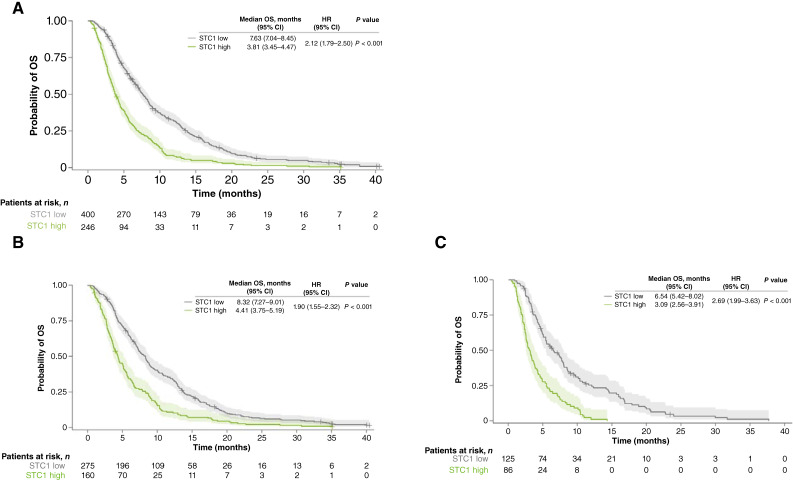
OS according to baseline STC1 plasma levels (optimized cut-off*) in patients with chemorefractory mCRC in the CORRECT study. **A,** Overall cohort (*N* = 646). **B,** Regorafenib cohort (*n* = 435). **C,** Placebo cohort (*n* = 211). *Optimized STC1 plasma levels: STC1 low: <1,436.87 pg/mL; STC1 high: ≥1,436.87 pg/mL.

In the overall population, patients with STC1-low levels had longer OS than patients with STC1-high levels. The median OS in the STC1-low cohort was 7.63 months (95% CI, 7.04–8.45) versus 3.81 months (95% CI, 3.45–4.47) in the STC1-high cohort (HR = 2.12, 95% CI, 1.79–2.50; *P* < 0.001; Fig. 2A). OS was significantly longer for patients with STC1-low levels than STC1-high levels in the regorafenib group (HR = 1.90, 95% CI, 1.55–2.32; *P* < 0.001; Fig. 2B) and the placebo group (HR = 2.69, 95% CI, 1.99–3.63; *P* < 0.001; Fig. 2C), supporting the prognostic value of this biomarker for OS.

We next explored the association of STC1 and key clinical and biological parameters with OS in patients from the CORRECT trial based on a multivariable Cox regression model including the following variables as covariates: treatment, age, ECOG performance status at baseline, liver metastases at baseline, number of metastatic sites, primary cancer site, *KRAS* mutational status, time since initial diagnosis, and the categorical biomarkers STC1, TIE2, and LDH (optimized cut-offs). Age and primary cancer site did not show an influence on OS, whereas STC1 plasma levels (HR = 1.35, 95% CI, 1.08–1.68; *P* = 0.009), treatment with regorafenib (HR = 0.71, 95% CI, 0.58–0.86; *P* < 0.001), ECOG performance status (HR = 1.53, 95% CI, 1.27–1.84; *P* < 0.001), and the presence of liver metastases (HR = 1.56, 95% CI, 1.21–2.02; *P* < 0.001) suggested an effect on OS (cross-validated C-index = 0.701).

### Predictive potential of STC1 in patients with chemorefractory mCRC treated with regorafenib or placebo in the CORRECT trial

**Figure 3:**
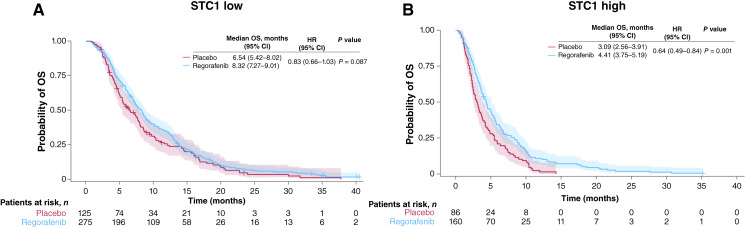
OS according to treatment (regorafenib vs. placebo) in patients with chemorefractory mCRC in the CORRECT trial in patients with (**A**) STC1-low levels or (**B**) STC1-high levels (optimized cut-off*). *Optimized STC1 plasma levels: STC1 low: <1,436.87 pg/mL; STC1 high: ≥1,436.87 pg/mL.

As STC1 expression is regulated during hypoxia and neoangiogenesis, we assessed whether STC1 protein levels could predict regorafenib efficacy compared with placebo using the patient population from the CORRECT trial. The treatment-interaction *P* value revealed a significant association between STC1 and regorafenib for OS (interaction *P* = 0.049; Supplementary Fig. S3), with patients benefitting from regorafenib treatment irrespective of the STC1 level. Overall, the median OS in the regorafenib group was 6.54 months (95% CI, 5.85–7.30) compared with 4.83 months (95% CI, 4.21–5.56) with placebo (HR = 0.75, 95% CI, 0.64–0.89; *P* < 0.001). In the STC1-low group, the median OS in the regorafenib group was 8.32 months (95% CI, 7.27–9.01) versus 6.54 months (95% CI, 5.42–8.02) with placebo, with an HR of 0.83, a 95% CI crossing 1 (CI, 0.66–1.03), and a nonsignificant *P* value of 0.087 (Fig. 3A). However, in the STC1-high group, the median OS with regorafenib was 4.41 months (95% CI, 3.75–5.19) versus 3.09 months (95% CI, 2.56–3.91) with placebo, with an HR of 0.64 (95% CI, 0.49–0.84) and a significant *P* value of 0.001 (Fig. 3B), indicating a potential predictive effect of STC1 on outcome. We also investigated the influence of LDH, another hypoxia-related biomarker, on regorafenib efficacy. In the CORRECT trial, the correlation (Spearman’s correlation coefficient) between LDH and STC1 was 0.59. A likelihood ratio test revealed no impact of LDH levels on regorafenib efficacy (*P* = 0.598). Regorafenib improved OS in both LDH-high (HR = 0.72, 95% CI, 0.56–0.93) and LDH-low (HR = 0.76, 95% CI, 0.61–0.95) groups (Supplementary Fig. S4A and S4B).

Due to the imbalances in ECOG performance status and liver metastases between STC1-low and -high groups, the additional predictive value of STC1 was evaluated based on a Cox proportional hazards model for OS. Adding STC1 to treatment, ECOG performance status 1, and presence of liver metastases increased the performance of the Cox model slightly from a C-index of 0.653 to 0.679. The likelihood ratio test also showed the add-on value of STC1 to ECOG performance status and presence of liver metastases (*P* < 0.001).

## Discussion

In this study, we showed that STC1 is a protein biomarker secreted at increased levels into the plasma in patients with mCRC refractory to chemotherapy. Furthermore, we identified an STC1 threshold that further defined a population of patients with high protein levels of STC1 who had poor prognosis in CORRECT. Importantly, all patients showed longer OS with regorafenib compared with placebo irrespective of STC1 levels.

We previously identified STC1 in a subset of patients with colorectal cancer expressing high levels of ANG2 (8). That analysis explored how angiogenic and stromal-related biomarkers measured in the plasma of patients with colorectal cancer might correlate with survival. Such investigations are mostly performed at the initiation of metastatic therapies in the first-line setting. ANG2 was identified as an independent adverse prognostic marker for OS in gene expression data from stage II/III colorectal cancer and in the plasma of patients with metastatic disease (8, 9). We showed that the ANG2-associated poor prognostic colorectal cancer subset was characterized by increased angiogenesis, myeloid cell infiltration, stromal organization, and resistance to chemotherapy. *STC1* was one of the major genes correlating with ANG2 and was demonstrated to be involved in molecular networks related to invasion and chemoresistance. STC1 has also been reported to be associated with gemcitabine resistance in pancreatic ductal adenocarcinoma (21). However, the potential clinical interest of STC1 in colorectal cancer has never been investigated until now.

Other studies have associated increased STC1 levels with a possible prognostic role in colorectal cancer based on mRNA levels in the tumor but not plasma protein levels in a randomized setting (22, 23). In the current study, we were able to determine that plasma STC1 levels were elevated in the setting of chemotherapy-resistant patients compared with samples taken at diagnosis of metastatic disease prior to any treatment. Indeed, although ANG2 levels are comparable in plasma of patients at diagnosis of their metastatic disease and after resistance to chemotherapy, STC1 levels are clearly higher following progression on several lines of chemotherapy. Furthermore, although ANG2 has a prognostic role in the first-line setting (9), we failed to identify a prognostic value of STC1 at the diagnosis of metastatic disease in the EPITOPES-CRC02 cohort. STC1 but not ANG2 influenced the clinical outcome of patients with mCRC previously exposed to chemotherapy. Analyses performed in our study support an additive value of STC1 to ECOG performance status, presence of liver metastases, and LDH on clinical outcomes. These observations confer a specific interest to STC1 as a biomarker in advanced disease settings.

Several observations support a potential role for STC1 in the progression of advanced carcinoma and as a potential biomarker for chemotherapy-resistant disease. STC1 expression has been demonstrated to be induced by hypoxia, a mechanism that promotes tumor angiogenesis, and to play a role in chemoresistance (21). Hypoxia-inducible factor-α promotes STC1 expression and subsequent PI3K/AKT downstream activation leading to pancreatic cancer chemoresistance in preclinical models (21). Another role of STC1 is in the suppression of oxidative stress and the prevention of mitochondrial damages. In this context, STC1 controls uncoupling protein 2 functions and activated protein kinase alpha to prevent hypoxia-mediated damages and apoptosis (24, 25). Therefore, STC1 expression by endothelial cells during hypoxia might promote a molecular network regulating cancer-associated fibroblast–tumor cell interactions, leading to colorectal cancer survival and progression. High STC1 plasma levels might contribute to the identification of patients with mCRC in whom such hypoxia-related molecular network is a major driver for their disease.

The OS benefit of regorafenib over placebo was numerically significant in the STC1-high group but not in the STC1-low group. One possible reason is that the STC1-low group is a good prognostic group in which patients may have been more likely to receive further treatment after regorafenib or placebo. In the CORRECT trial, treatment with regorafenib reduced the risk of death by 23% compared with placebo (HR = 0.77; ref. 5). The results described here show that the benefit from treatment with regorafenib compared with placebo increased to 36% in the STC1-high group (HR = 0.64 for OS), suggesting that the role of STC1 as a potential marker for treatment benefit with regorafenib compared with placebo in refractory colorectal cancer should be further assessed.

The limitations of this study include that it was a retrospective and exploratory analysis, leading to potential imbalances in the study groups, including ECOG performance status and liver metastases. The contribution and correlation of the prognostic effects of these factors require further analysis. In the absence of an established cut-off of STC1 protein plasma values in mCRC, we derived an optimized cut-off to best differentiate OS in the CORRECT cohort. This cut-off was used for prognostic and predictive analyses in CORRECT. Further confirmatory analyses on the optimal cut-off for maximum effect are required.

In conclusion, STC1 protein levels in plasma showed a prognostic effect on OS in patients with refractory mCRC, with STC1-high levels associated with poor outcomes. Although all patients benefitted from treatment with regorafenib, the poor prognostic group with STC1-high levels was observed to gain more OS benefit than the more prognostically favorable STC1-low group. Our results show that STC1 is a prognostic marker for OS in patients with chemorefractory mCRC and has a predictive potential of regorafenib versus placebo in terms of OS.

## Supplementary Material

Supplementary Data Table S1

Figure S1 Supplementary Figure 1

Figure S2 Supplementary Figure 2

Figure S3 Supplementary Figure 3

Figure S4 Supplementary Figure 4
