# Supplementary material for: Stanniocalcin 1 in Patients with Refractory Colorectal Cancer Treated with Regorafenib: A Post Hoc Biomarker Analysis of the TEXCAN and CORRECT Trials
Source: Cancer Res Commun. 2025 Feb 11;5(2):287–94. doi: 10.1158/2767-9764.CRC-24-0246 (PMC11811826; doi:10.1158/2767-9764.CRC-24-0246)
Supplement: Supplementary Data — Table S1 [file crc-24-0246_supplementary_data_suppst1.docx]

**Table S1.** Baseline STC1 protein levels in plasma in the overall patient population with evaluable samples from the CORRECT trial and according to study cohort (placebo; regorafenib).

|  | **N** | **STC1 (pg/mL)** | |
| --- | --- | --- | --- |
|  |  | **Mean (SD)** | **Median (range)** |
| Overall | 646 | 1473 (941) | 1211 (320–6568) |
| Placebo | 211 | 1501 (967) | 1233 (320–6223) |
| Regorafenib | 435 | 1460 (929) | 1181 (375–6568) |

SD, standard deviation.
