# Supplementary figures and images for: Stanniocalcin 1 in Patients with Refractory Colorectal Cancer Treated with Regorafenib: A Post Hoc Biomarker Analysis of the TEXCAN and CORRECT Trials
Source: Cancer Res Commun. 2025 Feb 11;5(2):287–94. doi: 10.1158/2767-9764.CRC-24-0246 (PMC11811826; doi:10.1158/2767-9764.CRC-24-0246)

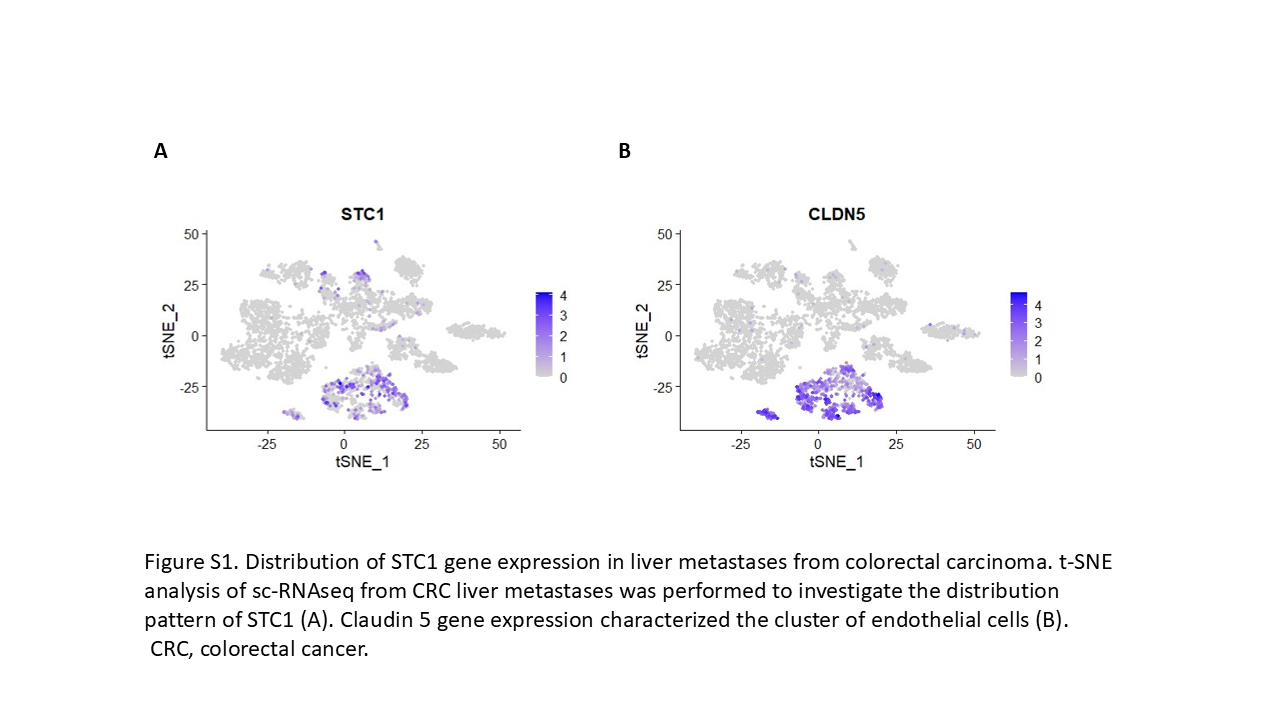

Supplement: Figure S1 — Supplementary Figure 1 [file crc-24-0246_figure_s1_suppsf1.png]
