# Supplementary material for: Stanniocalcin 1 in Patients with Refractory Colorectal Cancer Treated with Regorafenib: A Post Hoc Biomarker Analysis of the TEXCAN and CORRECT Trials
Source: Cancer Res Commun. 2025 Feb 11;5(2):287–94. doi: 10.1158/2767-9764.CRC-24-0246 (PMC11811826; doi:10.1158/2767-9764.CRC-24-0246)
Supplement: Figure S2 — Supplementary Figure 2 [file crc-24-0246_figure_s2_suppsf2.pptx]

## Slide 1
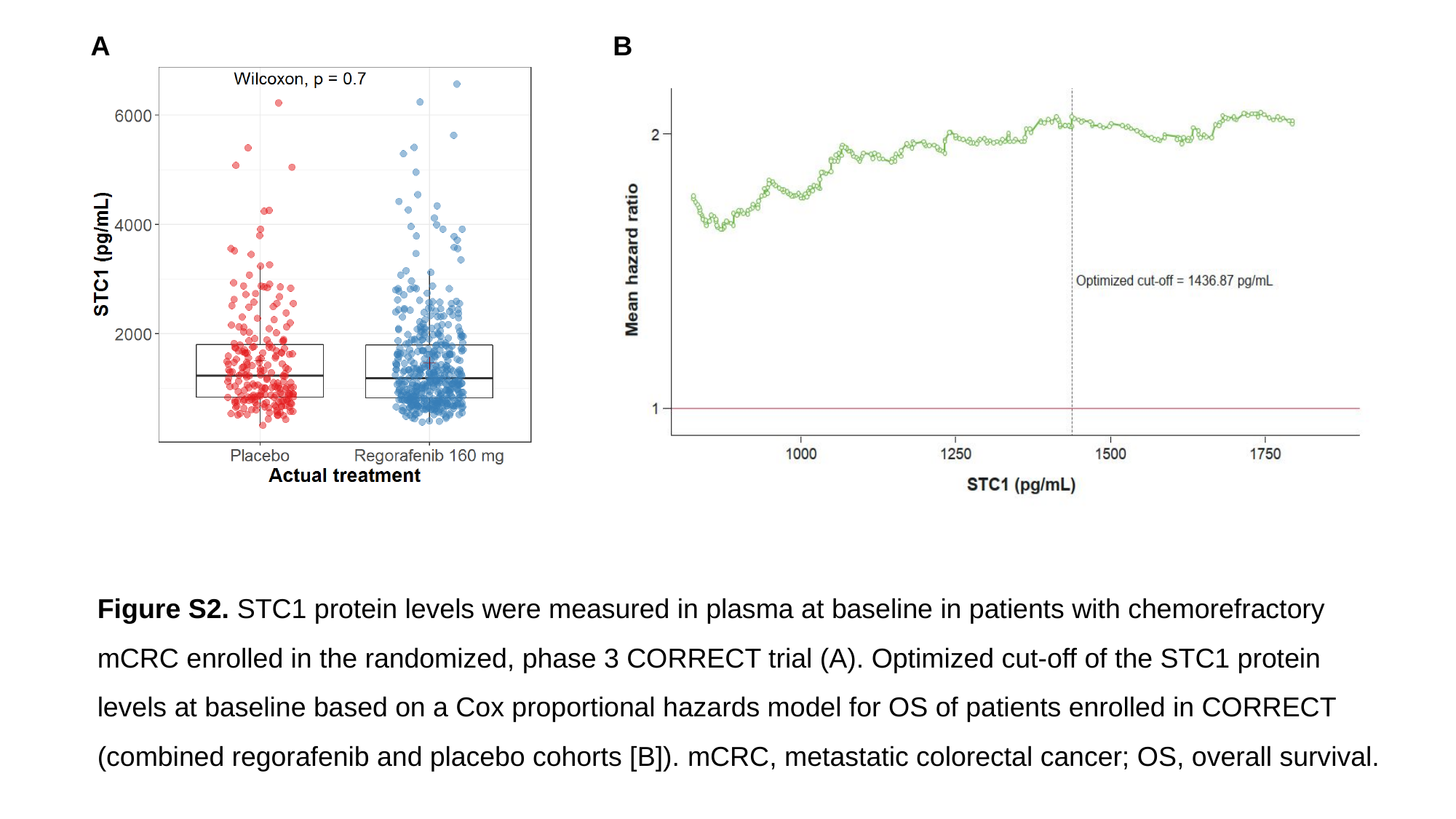

A
B
Figure S2. STC1 protein levels were measured in plasma at baseline in patients with chemorefractory mCRC enrolled in the randomized, phase 3 CORRECT trial (A). Optimized cut-off of the STC1 protein levels at baseline based on a Cox proportional hazards model for OS of patients enrolled in CORRECT (combined regorafenib and placebo cohorts [B]). mCRC, metastatic colorectal cancer; OS, overall survival.
