# Supplementary material for: Stanniocalcin 1 in Patients with Refractory Colorectal Cancer Treated with Regorafenib: A Post Hoc Biomarker Analysis of the TEXCAN and CORRECT Trials
Source: Cancer Res Commun. 2025 Feb 11;5(2):287–94. doi: 10.1158/2767-9764.CRC-24-0246 (PMC11811826; doi:10.1158/2767-9764.CRC-24-0246)
Supplement: Figure S3 — Supplementary Figure 3 [file crc-24-0246_figure_s3_suppsf3.pptx]

## Slide 1
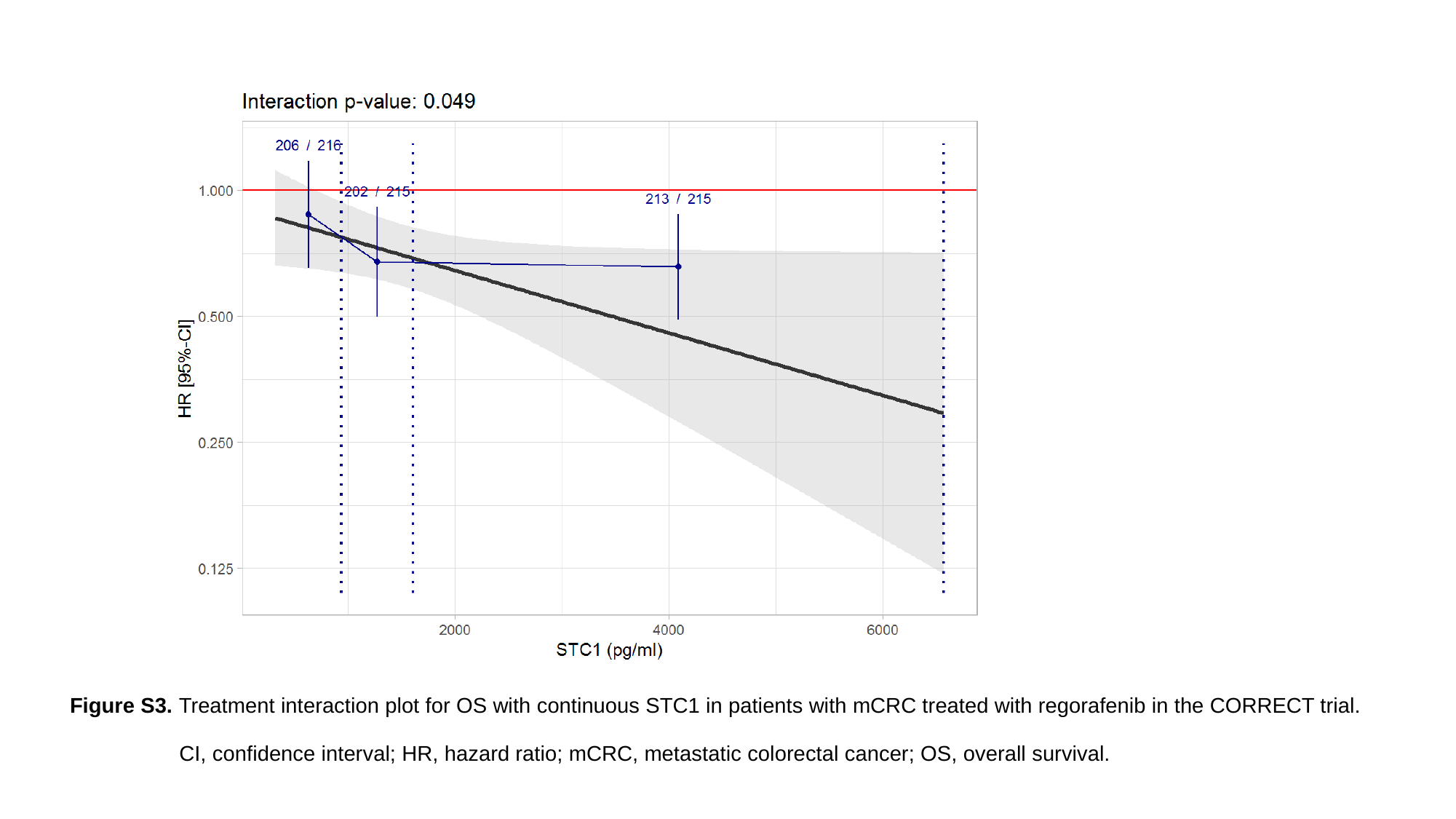

Figure S3. Treatment interaction plot for OS with continuous STC1 in patients with mCRC treated with regorafenib in the CORRECT trial.
CI, confidence interval; HR, hazard ratio; mCRC, metastatic colorectal cancer; OS, overall survival.
