# Supplementary material for: Stanniocalcin 1 in Patients with Refractory Colorectal Cancer Treated with Regorafenib: A Post Hoc Biomarker Analysis of the TEXCAN and CORRECT Trials
Source: Cancer Res Commun. 2025 Feb 11;5(2):287–94. doi: 10.1158/2767-9764.CRC-24-0246 (PMC11811826; doi:10.1158/2767-9764.CRC-24-0246)
Supplement: Figure S4 — Supplementary Figure 4 [file crc-24-0246_figure_s4_suppsf4.pptx]

## Slide 1
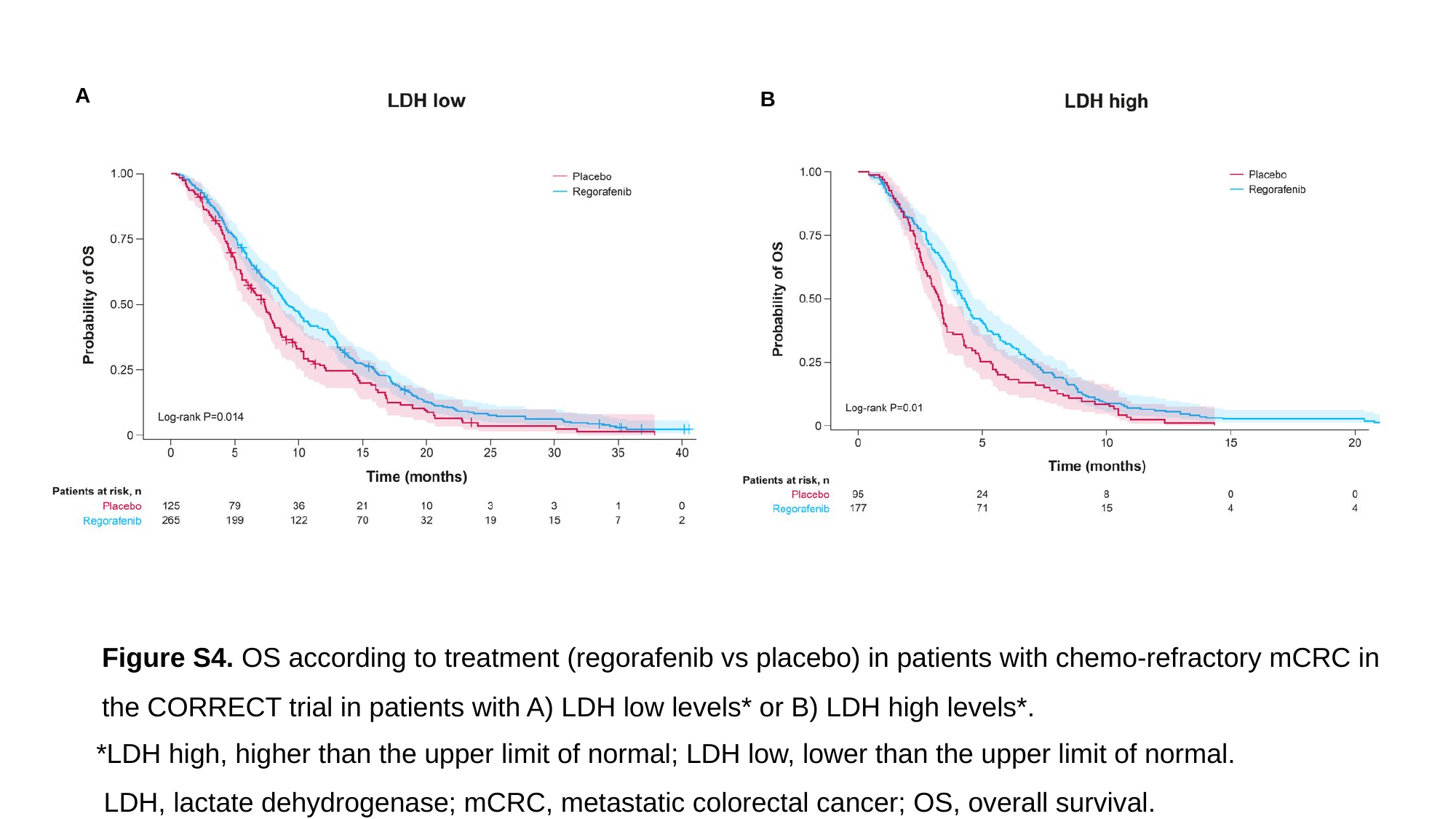

A
B
Figure S4. OS according to treatment (regorafenib vs placebo) in patients with chemo-refractory mCRC in the CORRECT trial in patients with A) LDH low levels* or B) LDH high levels*.
*LDH high, higher than the upper limit of normal; LDH low, lower than the upper limit of normal.
 LDH, lactate dehydrogenase; mCRC, metastatic colorectal cancer; OS, overall survival.
*LDH high, higher than the upper limit of normal; LDH low, lower than t
